# Peribacillus simplex and Klebsiella pneumoniae responsible for pyonephrosis with secondary psoas abscess: a case report

**DOI:** 10.1099/acmi.0.000911.v3

**Published:** 2025-01-14

**Authors:** Zakaria Malihy, Tilila Abassor, Yassine Ben Lahlou, Elmostafa Benaissa, Mariama Chadli

**Affiliations:** 1 Department of Bacteriology, Mohammed V Military Teaching Hospital/Faculty of Medicine and Pharmacy (University Mohammed V), Rabat, Morocco

**Keywords:** *Peribacillus simplex*, psoas abscess, pyonephrosis

## Abstract

Bacterial urinary tract infections (UTIs) are common, ranging from benign cystitis to complicated pyelonephritis, which can lead to severe complications such as pyonephrosis and sepsis. Pyonephrosis, characterized by the presence of pus in the renal cavities, often requires urgent urological intervention. We report a unique case of pyonephrosis with a psoas abscess caused by *Klebsiella pneumoniae *and *Peribacillus simplex *in a 64-year-old diabetic female patient. This is the first case of pyonephrosis caused by *P. simplex*. The patient presented with acute right lumbar pain, fever and altered consciousness. Imaging revealed severe right hydronephrosis, pyonephrosis and a perirenal phlegmon infiltrating the psoas with abscesses. Surgical drainage and nephrectomy were performed. Microbiological and proteomic analyses identified *K. pneumoniae* and *P. simplex*. This case highlights the importance of considering environmental bacteria like *P. simplex* in severe infections and ensuring rigorous protocols to avoid contamination. Successful management of pyonephrosis relies on prompt surgical drainage and appropriate antibiotic therapy based on culture results.

## Data summary

No data were reused or generated in this study.

## Introduction

Bacterial urinary tract infections (UTIs) are a frequent cause of community- and healthcare-associated infections. These heterogeneous conditions can range from simple benign acute cystitis or pyelonephritis to complicated acute pyelonephritis facilitated by underlying conditions. Complications can include abscess formation progressing to pyonephrosis, haematogenous or contiguous dissemination potentially leading to sepsis with secondary foci or even septic shock, threatening the patient’s life.

Pyonephrosis is defined as the presence of a purulent collection in the renal cavities with partial or total destruction of the renal parenchyma associated with significant perinephritis [1]. It is a rare urological emergency in adults, where the variability of clinical signs makes diagnosis even more challenging [2]. The aetiology is primarily bacterial, with the most commonly implicated pathogens being enterobacteria, mainly *Escherichia coli* and *Klebsiella pneumoniae* [3].

We report a unique case of pyonephrosis with a secondary psoas abscess due to a strain of *K. pneumoniae* associated with a strain of *Peribacillus simplex *in a 64-year-old diabetic female patient. To our knowledge, this is the first published case of pyonephrosis due to *P. simplex* and the third case of *P. simplex* infection in the world.

## Case report

The patient is a 64-year-old woman with a medical history of poorly controlled type II diabetes under oral antidiabetics. She presented to our facility with a sudden onset of intense acute right lumbar pain, fever and altered consciousness. The initial clinical examination revealed tachycardia and polypnea.

Emergency biological workup showed a significant increase in C-reactive protein (246 mg l^−1^), hyperleucocytosis (18.2×10⁹ l^−1^) and inflammatory anaemia (haemoglobin: 9.5 g dl^−1^, mean corpuscular haemoglobin concentration: 29 g dl^−1^ and mean corpuscular volume: 70 fl). Renal assessment revealed elevated creatinine levels (47 mg l^−1^) and decreased glomerular filtration rate (10 ml min^−1^ 1.73 m^−2^).

**Fig. 1.**
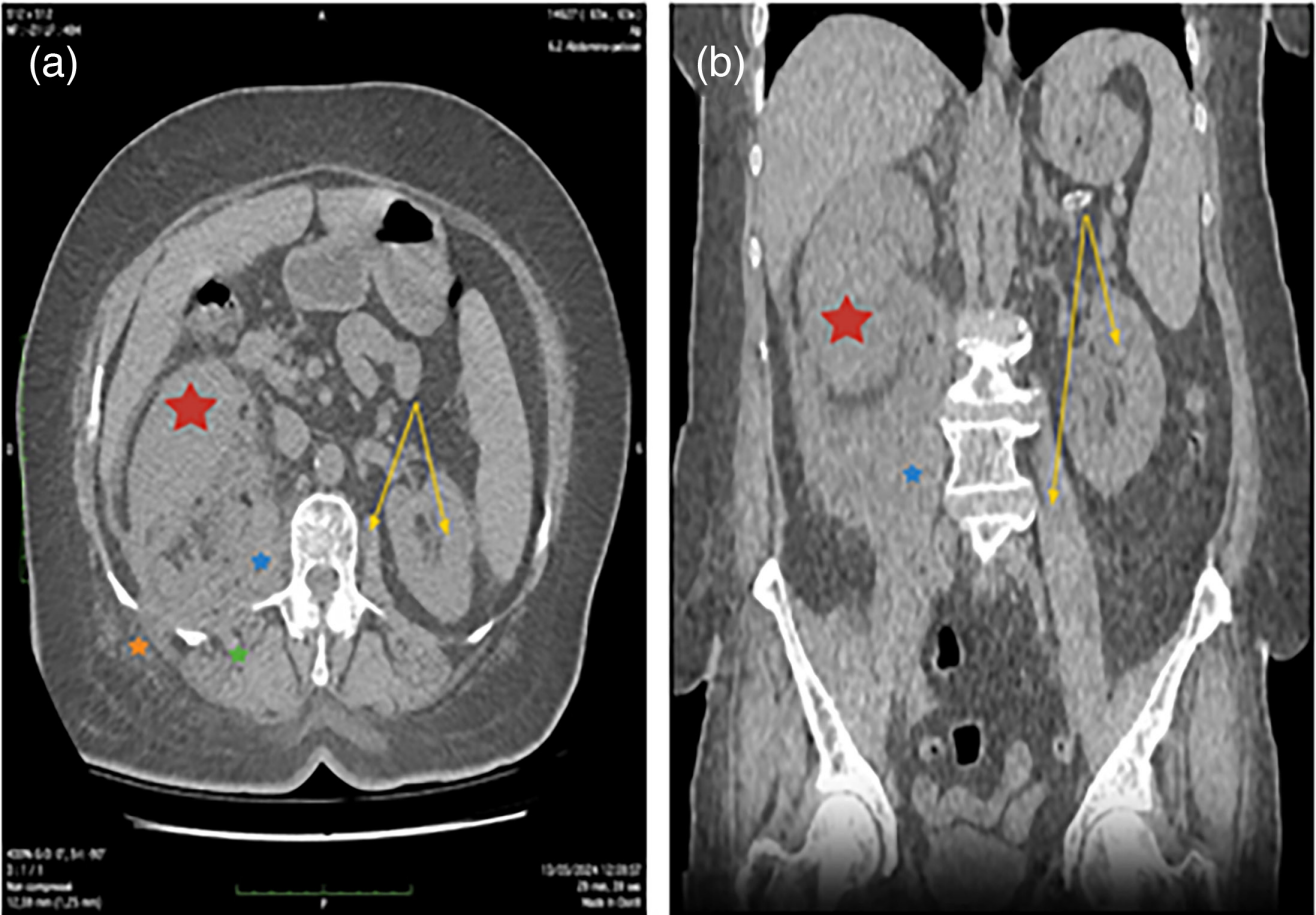
Axial (**a**) and coronal (**b**) views of a CT scan showing a destroyed right kidney (red star) with disappearance of the pyelocaliceal cavities (pyonephrosis) and perirenal phlegmon extending to the psoas muscle (blue star), the paravertebral muscles (green star) and the muscles of the posterior abdominal wall through the intercostal spaces (orange star). For comparison, the yellow arrow represents the non-infiltrated contralateral left side (kidney and psoas muscle).

Imaging studies were rapidly initiated to determine the nature of the lesions and assess their extent. Renal and bladder ultrasound scan revealed severe unilateral right uretero-hydronephrosis, pyonephrosis and bladder lithiasis. An abdominal-pelvic computed tomography (CT) scan without and with contrast (Fig. 1) showed findings consistent with right pyeloureteritis complicated by pyonephrosis and a significant perirenal phlegmon infiltrating the homolateral psoas muscle with two abscesses within the muscles of the posterior abdominal wall and right paravertebral muscles.

Given this scenario of purulent retention in the pyelocaliceal cavities, surgical drainage of the renal cavities was deemed necessary before performing a nephrectomy. A 3 cm lumbar incision was made. Dissection proceeded to the abscess cavity in the retroperitoneal space, crossing the psoas muscle, where the abscess was drained using a Redon drain through a counter-incision in the right flank. The pus collected was immediately sent to the laboratory for cytobacteriological analysis. In addition, one set of blood cultures in aerobic and anaerobic media was performed.

Direct examination of the pus with Gram staining showed altered polymorphonuclear cells associated with a polymorphic bacterial flora consisting of numerous Gram-negative and Gram-positive bacilli. The pus was inoculated into brain–heart infusion (BHI) broth, blood agar and chocolate agar with Polyvitex incubated under a CO_2_-enriched atmosphere. Additionally, blood agar supplemented with inhibitors [nalidixic acid–colistin (NAC)] and Schaedler agar were inoculated and incubated in an anaerobic jar with an anaerobic generator system. Aerobic cultures were observed every 24 h, while anaerobic cultures were observed every 48 h. The BHI broth was subcultured after 24 h of incubation onto NAC blood agar and chocolate agar was incubated under the same conditions.

After 24 h of incubation, a biomorphic culture of large, white, shiny mucoid colonies and small, dry whitish colonies was observed on aerobic media. Anaerobic cultures showed a monomorphic culture of large, white, shiny mucoid colonies. The same results were found on subcultures except for the NAC agar incubated aerobically, which showed a monomorphic aspect with small, dry whitish colonies.

The aerobic and anaerobic blood culture bottles became positive at 8 and 12 h, respectively. The direct Gram stain examination and the culture on blood agar supplemented with NAC and chocolate agar with Polyvitex showed the same results as the pus sample.

Identification was performed using MALDI-TOF mass spectrometry. The large, white, shiny mucoid colonies corresponded to *K. pneumoniae*, while the small dry colonies corresponded to *P. simplex*.

Antibiotic susceptibility testing was performed using the disc diffusion method according to the Antibiogram Committee of the French Society for MicrobiologyAC-FSM 2023 recommendations. The *K. pneumoniae* strain produced an extended-spectrum beta-lactamase (ESBL) conferring resistance to aminopenicillins and cephalosporins while remaining sensitive to cephamycins, penicillin–inhibitor combinations, co-trimoxazole, carbapenems and fluoroquinolones. The *P. simplex* strain was broadly sensitive to tested antibiotics, with critical diameters for *Bacillus* spp. being used for result interpretation. The strain was sensitive to carbapenems, linezolid, vancomycin and norfloxacin and sensitive at high exposure to ciprofloxacin and levofloxacin but resistant to erythromycin and clindamycin. Table 1 summarizes the antibiotic resistance pattern of *P. simplex* and *K. pneumoniae*.

**Table 1.** Antibiotic resistance pattern of *P. simplex* and *K. pneumoniae*

	*P. simplex*	*K. pneumoniae*
Aminopenicillins	N/T	R
Cephalosporins	N/T	R
Cephamycins	N/T	S
Penicillin–inhibitor combinations	N/T	S
Carbapenems	S	S
Co-trimoxazole	N/T	S
Levofloxacin	I	S
Linezolid	S	n/a
Vancomycin	S	n/a
Norfloxacin	S	n/a
Erythromycin and clindamycin	R	n/a

R = resistant, S = sensitive, I = intermediate, N/A = not applicable, N/T = not tested.

After drainage, the patient was treated empirically with ceftriaxone and amikacin for 72 h. Following the antibiogram results, the patient received high-dose levofloxacin, resulting in significant clinical and biological improvement, including a decrease in inflammatory parameters and resolution of sepsis signs. Clinical stabilization allowed the patient to be discharged with oral antibiotics to complete a 14-day treatment course.

## Discussion

The advent of new taxogenomic methods has led to the reclassification of many species and the emergence of new genera. Previously classified within the genus *Bacillus* by Meyer and Gottheil (1901), *P. simplex* has been described as an environmental organism commonly found in soil [4,5] and deteriorated mural paintings [6,7]. Data on the geographical distribution of *P. simplex* are limited. *Bacillus maroccanus*, which is classified under *B. simplex* based on the current species definition [8], was originally identified in warm arid soils in Morocco [9]. Other strains have also been discovered in deteriorated mural paintings located in Spain (Carmona necropolis) and Germany [7].

To date, two cases of *P. simplex* infections have been reported: a wound infection [10] and a brain abscess [11]. This study reports the first case of pyonephrosis involving a strain of *P. simplex* and *K. pneumoniae*. The concurrent presence of *P. simplex* and *K. pneumoniae*, leading to secondary psoas abscess formation, highlights a unique microbial interaction not well documented in the literature.

Pyonephrosis is an accumulation of pus in the renal parenchyma causing its obstruction. It is an aggressive condition associated with intense and destructive renal inflammation accompanied by potentially fatal septicaemia. It primarily affects adults (70% of cases) with a mean age between 42 and 51 years, with a higher incidence in women, and most commonly affects the right kidney [3]. The epidemiological profile aligns with our patient’s case.

Like any abscess formation, pyonephrosis typically presents with fever, chills and pain, notably in the lumbar region or abdomen (flank) but can be asymptomatic [2]. Urinary source bacteraemia causes sepsis signs, including haemodynamic instability, leucocytosis, inflammatory anaemia and renal failure [3]. Our patient exhibited the typical clinical presentation along with urosepsis.

The main risk factors are urinary tract obstruction and poorly controlled diabetes [2,12, 13]. An obstruction cause, particularly kidney stones, is found in over 70% of cases [13,19]. These were indeed the risk factors present in our patient. A major risk of developing pyonephrosis is immunosuppression [12]. Xanthogranulomatous pyelonephritis is a less common cause.

The most common complication is peritonitis, while the occurrence of a psoas abscess is rare [2]. Our patient presented with a secondary psoas abscess. The iliopsoas muscle is in close contact with the kidney, its vessels and the ureter. This anatomical relationship predisposes the iliopsoas to secondary abscesses from the extension of infection from adjacent sites. Another rare complication reported was pleural empyema [15].

Radiological examinations are crucial for the immediate diagnosis and management of this pathology and its complications, given that death can rapidly occur from septic shock. Ultrasound and CT scans are the preferred imaging methods for diagnosing and staging this pathology in emergency settings.

Pus culture is the gold standard for the aetiological diagnosis of pyonephrosis. Urine cultures frequently yield negative results. Gram-negative bacteria are most commonly associated with this condition, particularly *E. coli* (30%) and *K. pneumoniae* (19%) [20]. In our case, we have found the unique and atypical association of *P. simplex* and *K. pneumoniae*. Other atypical micro-organisms that have been reported include *Candida albicans* [13], Group B *Streptococcus* [14], *Proteus mirabilis* [15], *Salmonella* species [16,18] and associations between anaerobes and *Enterobacteriaceae* [12,17].

*P. simplex* is a Gram-positive bacterium capable of sporulation with an aerobic metabolism [6]. Some *P. simplex* strains (N65.1 and N58.2) produce toxins capable of damaging immune cells by inhibiting mitochondrial activity, as well as haemolytic and non-haemolytic toxins [10,21, 22]. Haemolysis releases nutrients, allowing the bacterium to survive and cause cellular damage, thus accessing deep tissues. Although there are limited data in the literature describing the virulence of *P. simplex*, the cytotoxic properties of these toxins must be considered in our patient’s case. To date, *P. simplex* has been isolated in a stool sample from a patient in Saudi Arabia, as part of a culturomic study aimed at identifying all species within the gut microbiota [23]. It is well established that UTIs often originate from a patient’s endogenous flora, influenced by host-specific factors and characteristics of the pathogenic strain [24]. In our case, *P. simplex* gut may result from environmental exposure, potentially through contact with contaminated soil, dust or surfaces, which could lead to digestive and possibly urethral colonization. This could allow the pathogen to subsequently migrate to the bladder and then the right kidney. A synergistic microbial effect may have occurred; however, it has not been documented in the literature for these two strains.

Currently, data on the antimicrobial sensitivity of this organism are limited. Our strain showed isolated resistance to macrolides, unlike the strain isolated in a wound infection that was sensitive to imipenem, clindamycin and ciprofloxacin [10].

*K. pneumoniae*’s pathogenicity is due to numerous virulence factors, allowing it to evade the immune response (polysaccharide capsule), adhere to mucous membranes with biofilm formation (adhesins and slime secretion) and induce toxic shock (LPS). A siderophore system ensures its survival in a hostile environment through competitive iron capture [25]. Multi-drug resistance, particularly through ESBL and carbapenemase production, complicates therapeutic management. In our case, both *K. pneumoniae* and *P. simplex* strains showed susceptibility to levofloxacin, which guided the choice of this antibiotic.

The treatment of pyonephrosis involves surgery followed by targeted antibiotic therapy based on antibiogram results. Surgical options include percutaneous pus drainage, retrograde ureteral drainage with a double J stent and, in extreme cases, nephrectomy. In our case, a right nephrectomy was necessary due to irreversible loss of renal function.

## Conclusion

This case highlights a rare occurrence of pyonephrosis caused by *P. simplex* in conjunction with *K. pneumoniae*, leading to a secondary psoas abscess.

The identification of *P. simplex* in a human infection context underscores the importance of considering atypical pathogens in complex pyonephrosis cases, especially when the initial urine cultures return negative. Rigorous steps are essential to rule out contamination and confirm accurate pathogen identification in such rare cases. Here, the sample was obtained from a sterile site, with strict aseptic protocols both in collection and laboratory processing, confirming the involvement of *P. simplex* in pyonephrosis.

The successful management of this case was achieved by both thorough microbiological testing and appropriate imaging and timely surgery, all of which guided effective treatment. Generally, managing pyonephrosis requires urgent surgical drainage combined with targeted antibiotic therapy based on culture and susceptibility results.

The development of secondary complications like a psoas abscess emphasizes the importance of prompt imaging in pyonephrosis cases. Clinicians should remain vigilant for uncommon pathogens, particularly in patients with risk factors such as urinary obstruction and compromised immunity.

This case underscores the need for further research into the epidemiology and pathogenicity of *P. simplex* and similar organisms in human infections. Additional studies investigating potential environmental exposure routes, mechanisms of colonization and effective treatment protocols for rare pathogens in pyonephrosis or other severe infections are warranted to improve clinical outcomes for similar cases.

## Abbreviations

BHI: brain–heart infusion
ESBL: extended-spectrum beta-lactamase
NAC: nalidixic acid–colistin
UTIs: urinary tract infections
